# Multi-factorial barriers and facilitators to high adherence to lung-protective ventilation using a computerized protocol: a mixed methods study

**DOI:** 10.1186/s43058-020-00057-x

**Published:** 2020-07-28

**Authors:** Andrew J. Knighton, Jacob Kean, Doug Wolfe, Lauren Allen, Jason Jacobs, Lori Carpenter, Carrie Winberg, Jay G. Berry, Ithan D. Peltan, Colin K. Grissom, Raj Srivastava

**Affiliations:** 1 Implementation Science Research, Healthcare Delivery Institute, Intermountain Healthcare, 5026 South State Street, 3rd Floor, Murray, UT 84107 USA; 2 Population Health Sciences, University of Utah School of Medicine, 295 Chipeta Way, Salt Lake City, UT 84108 USA; 3 Best Practice Implementation, Healthcare Delivery Institute, Intermountain Healthcare, 5026 South State Street, 3rd Floor, Murray, UT 84107 USA; 4 Pulmonary and Critical Care Research, Intermountain Healthcare, 5121 S Cottonwood St, Murray, UT 84107 USA; 5 Complex Care, Division of General Pediatrics, Boston Children’s Hospital, Harvard Medical School, 300 Longwood Avenue, Boston, MA 02115 USA; 6 Division of Pulmonary and Critical Care Medicine, Department of Internal Medicine, University of Utah School of Medicine, Salt Lake City, USA; 7 Division of Pulmonary and Critical Care Medicine, Intermountain Healthcare, 5121 S Cottonwood St, Murray, UT 84107 USA; 8 Intermountain Healthcare, 5121 S Cottonwood St, Murray, UT 84107 USA; 9 Healthcare Delivery Institute, Intermountain Healthcare, 5026 South State Street 3rd Floor, Murray, UT 84107 USA; 10 Department of Pediatrics, University of Utah School of Medicine, 295 Chipeta Way, Salt Lake City, UT 84108 USA

**Keywords:** Acute respiratory distress syndrome (ARDS), Mechanical ventilation, Computerized protocol, Clinical decision support, Implementation, Barriers and facilitators, Mixed methods

## Abstract

**Background:**

Lung-protective ventilation (LPV) improves outcomes for patients with acute respiratory distress syndrome (ARDS) through the administration of low tidal volumes (≤ 6.5 ml/kg predicted body weight [PBW]) with co-titration of positive end-expiratory pressure and fraction of inspired oxygen. Many patients with ARDS, however, are not managed with LPV. The purpose of this study was to understand the implementation barriers and facilitators to the use of LPV and a computerized LPV clinical decision support (CDS) tool in intensive care units (ICUs) in preparation for a pilot hybrid implementation-effectiveness clinical trial.

**Methods:**

We performed an explanatory sequential mixed methods study from June 2018 to March 2019 to evaluate the variation in LPV adherence across 17 ICUs in an integrated healthcare system with > 4000 mechanically ventilated patients annually. We analyzed 47 key informant interviews of ICU physicians, respiratory therapists (RTs), and nurses in 3 of the ICUs using a qualitative content analysis paradigm to investigate site variation as defined by adherence level (low, medium, high) and to identify barriers and facilitators to LPV and LPV CDS tool use.

**Results:**

Forty-two percent of patients had an initial set tidal volume of ≤ 6.5 ml/kg PBW during the measurement period (site range 21–80%). LPV CDS tool use was 28% (site range 6–91%). This study’s main findings revealed multi-factorial facilitators and barriers to use that varied by ICU site adherence level. The primary facilitator was that LPV and the LPV CDS tool could be used on all mechanically ventilated patients. Barriers included a persistent gap between clinician attitudes regarding the use of LPV and actual use, the perceived loss of autonomy associated with using a computerized protocol, the nature of physician-RT interaction in ventilation management, and the lack of clear organization measures of success.

**Conclusions:**

Variation in adherence to LPV persists in ICUs within a healthcare delivery system that was an early adopter of LPV. Potentially promising strategies to increase adherence to LPV and the LPV CDS tool for ARDS patients include initiating low tidal ventilation on all mechanically ventilated patients, establishing and measuring adherence measures, and focused education addressing the physician-RT interaction. These strategies represent a blueprint for a future hybrid implementation-effectiveness trial.

Contributions to the literature

- Research has shown that lung-protective ventilation (LPV) improves outcomes for patients with acute respiratory distress syndrome and is not consistently applied in intensive care settings. Clinical decision support (CDS) tools are being evaluated as one mechanism to standardize LPV use.
- While CDS tools may assist in normalizing the use of LPV, we found multi-factorial barriers and facilitators to LPV and to LPV CDS tool use in an organization involved in early LPV studies.
- These findings contribute to recognized gaps in the literature, including detailing barriers to LPV and CDS tool use and describing theory-informed, tailored implementation strategies.

## Background

Acute respiratory distress syndrome (ARDS) occurs when an acute lung injury (e.g., pneumonia, sepsis, trauma) causes bilateral non-cardiogenic pulmonary edema and hypoxemic respiratory failure requiring mechanical ventilation [1]. An international study in 50 countries found that 10% of patients admitted to the intensive care unit (ICU) and 23% of mechanically ventilated patients had ARDS [2]. During the 2019 novel coronavirus (COVID-19) pandemic, 20–42% of hospitalized patients and 67–85% of patients admitted to the ICU will develop ARDS [3]. ARDS is associated with high morbidity, mortality, and healthcare cost [2, 4, 5]. Lung-protective ventilation (LPV), which combines low tidal volume ventilation (LTVV) with step-wise co-titration of positive end-expiratory pressure (PEEP) and the fraction of inspired oxygen (FiO_2_), improves outcomes for patients with ARDS in clinical studies [5, 6] and is recommended by the American Thoracic Society (ATS) Clinical Practice Guidelines [7, 8] as well as in other recent reviews [9, 10].

Barriers to consistent use of LPV persist and have hindered progress toward improved ARDS outcomes [2]. General agreement exists among physicians, respiratory therapists (RTs), and nurses that LPV is warranted for patients with ARDS, although studies demonstrate a substantial divide between belief and actual practice [11]. Identified barriers to the use of LPV include physician’s ability to recognize ARDS in a timely fashion [12, 13], lack of written protocols [14, 15], lack of concordance with clinician perceptions of patient needs [11, 12, 14], and perceptions by nurses and RTs that LTVV is more labor-intensive and that present staffing is inadequate to achieve full adherence [11, 14].

Intermountain Healthcare (Intermountain) was involved in early ARDS network studies [5] and demonstrated high adherence at one hospital site, but high system-wide adherence was not attained across all sites. We recently deployed an electronic medical record (EMR)-integrated LPV clinical decision support (CDS) tool to the 17 ICUs in our system designed to standardize LPV practice across all sites and increase overall LPV adherence. We conducted a mixed methods study to identify barriers to utilization of LPV and the LPV CDS tool in patients with ARDS in preparation for a pilot hybrid implementation-effectiveness trial (NCT03984175) designed to increase adherence to LPV and the LPV CDS tool in ARDS patients.

## Methods

We performed an explanatory, sequential mixed methods study (quant -> QUAL) from June 2018 to March 2019 to (1) measure the variation in adherence to LPV and to the use of the LPV CDS tool across 17 ICU sites and (2) understand, through qualitative interviews, the reasons for variation and to identify implementation barriers and facilitators to LPV and LPV CDS tool in routine practice for patients with ARDS at three of those ICU sites [16, 17]. The research protocol was approved by the Intermountain Healthcare Institutional Review Board (IRB# 1050867). We adhered to published best practices for reporting of mixed methods studies [17, 18] and qualitative research [19].

Intermountain Healthcare’s 17 ICUs are part of an integrated, 24-hospital system that includes frontier hospitals, tertiary care centers, and a children’s hospital. Across the system, roughly 4300 adult patients are ventilated annually. Approximately 20% of these ventilated patients have ARDS [2, 20]. To improve the use of LPV in mechanically ventilated patients, a team of critical care clinicians and researchers developed a novel computerized ventilation protocol integrating open-loop CDS within the EMR. In open-loop CDS systems, a clinician may receive proposed setting changes from the CDS tool, but the clinician makes the final determination each time regarding the actual setting changes. The tool was deployed across Intermountain hospitals in parallel with the deployment of a new system-wide EMR (Cerner Corporation, Kansas City, MO) that began in April 2016 and was completed in July 2017. The tool integrates four different open-loop computerized protocols: an LTVV protocol, an oxygenation protocol targeting normal oxygenation and normal or high PEEP strategies, a weaning assessment, and a spontaneous breathing protocol.

To initiate the use of the open-loop protocols, physicians must place an electronic order for each protocol component. Once ordered, the RT measures patient height and sets parameters for tidal volume, respiratory rate, FiO_2_, and PEEP. The protocol order does not specify the starting settings. However, if the RT does not use an LTVV setting, the protocol will give instructions to the RT to move to an LTVV setting. An arterial blood gas (ABG) is obtained within 1 h. Oxygenation and ventilation protocols are then run, generating instructions for the RT to adjust or maintain FiO_2_, PEEP, tidal volume, and respiratory rate. The RT must accept or reject each instruction and, if instructions are rejected, provide a reason. The ventilation protocol is intended to run each time an ABG result is received. The oxygenation protocol is intended to be run after ABG results and also every 2 h based on oxygen saturation measured from pulse oximetry (SpO_2_) during each RT ventilator assessment.

During initial deployment, LPV CDS tool implementation strategies included didactic education, communications, audit and feedback to physicians and RTs, and executive leadership emphasis on compliance. However, some ICUs were not achieving consistent adherence to the initial set tidal volumes ≤ 6.5 ml/kg PBW or consistent utilization of LPV CDS tools in mechanically ventilated patients.

### Estimating adherence by site

We used two provisional measures to explore the variation in adherence by site. Using an encounter cohort of ICU-admitted adult patients (age ≥ 18 years) receiving invasive mechanical ventilation, we calculated the percentage of patient ICU encounters that were treated with an initial set tidal volume ≤ 6.5 ml/kg PBW. For the second measure, we calculated the percentage of patient ICU encounters that were treated using at least one of the four protocols in the LPV CDS tool during the stay. The cohort excludes patients who died on the day of admission. The cohort represents data from a restricted study time period from the IMPROVENT clinical trial (NCT03225807). The measurement period for cohort identification began on the date the LPV CDS tool was deployed at each site (ranging from April 2016 to July 2017) and ended on October 31, 2017. The calculations were made from data queried from the Intermountain EMR systems. Spearman’s rank test was used to observe any initial correlation between the two measures. A two-sample test of proportions was used to explore the differences in both measures at the aggregate level by hospital type.

### Tailored interview guide development

We developed an interview guide using a deductive, multi-method approach: a scoping review [21–24] to examine the barriers and facilitators to the use of LPV and the LPV CDS tool and interventions to improve adherence; a technical expert panel that included 4 critical care physicians, 2 hospitalists/health services researchers, 2 ICU nurse managers, 1 emergency department (ED) physician, 1 respiratory therapist (RT), and 1 implementation scientist, to identify already known or suspected barriers to implementation (simultaneous triangulation) [25]; and categorization and summary of findings according to the Consolidated Framework for Implementation Research (CFIR) [26, 27] by two experienced implementation scientists (AK, RS). This approach is consistent with the efforts to develop contextual implementation frameworks for complex system interventions [28].

CFIR constructs identified as relevant through both the scoping review and by the multi-disciplinary expert panel formed the theoretical basis for the interview guide questions. Common, relevant CFIR domains (and related constructs) identified for both LPV and the LPV CDS tool included the individual (knowledge and beliefs), intervention (relative advantage, adaptability, design quality, and packaging), and the inner setting (learning climate, compatibility, and available resources). LPV also included intervention (evidence strength and quality) and inner setting domains (tension for change, relative priority, and leadership engagement). The LPV CDS tool also included individual (individual stage of change), intervention (complexity), and inner setting domains (goals and feedback). No constructs were initially identified as relevant from the external setting and implementation domains. Validated interview questions related to the selected CFIR constructs were then drawn from the “Barriers to Physician Adherence to Practice Guidelines” model and adapted for interdisciplinary interviews to guide the assessment of knowledge, attitudes, and behaviors of sites and individual caregivers regarding LPV use [29]. To assess the barriers and facilitators of LPV CDS tool use, validated questions were adapted from the “Unified Theory of Acceptance and Use of Technology” (UTAUT) to explain user intentions to use an information system and subsequent usage behavior [30]. Upon completion, the interview guide and questions were reviewed by the Intermountain lead critical care physician (CG) and respiratory therapist (CW) for clarity and relevance.

### Key informant semi-structured interviews

To understand the primary reasons for the variation in adherence at each site using a grounded theory approach [17, 31], key informant semi-structured interviews were conducted using the interview guide with clinicians at Intermountain ICUs. To ensure an information-rich sample [32], three pilot ICU sites were selected by the research team from the quantitative analysis. The team used a stratified purposeful sampling approach [33] based upon site adherence ranking (high-medium-low), ICU type (medical/surgical, respiratory, cardiac, neurologic, and thoracic), and local leadership support. Local leadership support was determined through conversations between the system ICU leader and local site ICU leaders. Given the study focus on patients with ARDS, site selection was limited to medical/surgical, trauma, and respiratory ICU sites.

A two-person team of trained, experienced qualitative researchers (AJK, DW) conducted the key informant interviews with a purposive sample of 10–20 key informants using a role-based criterion. Interview participant roles included the ICU physician director, critical care physicians, ICU RT manager, ICU RTs, and ICU nurses with the goal to achieve uniform participation at each site across roles, with an option to sample additional roles as needed. For roles that were not limited to a single individual at a site (intensivists, RTs, and nurses), interviews continued until thematic saturation was reached (no new ideas emerged during three consecutive interviews for a particular role at a site) [34]. Each interview was 30 min. While adherence data was available to individual sites, the investigators did not present site adherence data in the discussion. Research funds were made available on the day of each site visit at all three sites to schedule additional clinical resources to ensure patient coverage. Participants at each site were invited to participate on the day of the site visit by the local ICU director and RT manager via email or direct conversation, subject to availability. Efforts were made to identify individuals within each role that varied in terms of years of their experience and attitudes and beliefs regarding LPV and the LPV CDS tool. Preliminary assessments of attitudes and beliefs were based upon the local ICU director or site RT manager’s experience working with each clinician. The qualitative research team met frequently during each site visit to review interview data and to assess thematic saturation more generally and to identify additional interview needs by role. At the end of each site visit, emerging themes and ideas were summarized and shared with system and site clinical leaders and were used to identify follow-up interviews to address the gaps in understanding.

### Qualitative data analysis

A hybrid qualitative content analysis paradigm was applied to interview data, incorporating both directed and open-iterative methods to provide a reflexive approach to identify barriers and facilitators to high adherence to the use of LPV and the LPV CDS tool [35]. A study investigator and experienced qualitative researcher (AK) organized a preliminary codebook by interview guide question number as the unit of analysis. (The interview guide was originally organized deductively by relevant CFIR construct.) Two research assistants were trained by the study co-investigator on both the clinical and non-clinical aspects of the study, including specific training on the use of the preliminary codebook. The study investigator (AK) then read and conducted line-by-line coding of a preliminary sample of five interviews using the preliminary codebook, allowing for the open coding of new or emerging themes not already captured. The five transcripts were divided between the two research assistants who separately read and independently conducted line-by-line coding using a similar approach. For each coded transcript, the study co-investigator and the independent reviewers compared the results, agreeing on the name and definition of each code. This codebook was then used to assist the researchers in the analysis of the remaining interview data.

The study team followed a similar process for the remaining interviews. During the coding process, the coding team met frequently to identify and agree on new or emerging concepts not captured in the current codebook and recoded prior transcripts accordingly. For each coded transcript, the arbitration for discordant coding was done through a discussion between the study co-investigator and the assigned independent reviewer until consensus was reached. Reported implementation barriers and facilitators were then summarized according to the levels of the CFIR framework overall, by site and by clinical role [26]. All coding was done in Atlas.ti version 8.0 (Scientific Software Development GmbH, Berlin, Germany).

## Results

The provisional estimate of the initial set tidal volume ≤ 6.5 ml/kg PBW was 42% system-wide. ICU-level adherence estimates ranged from 21 to 80% (Table 1). The estimated LPV CDS tool utilization was 28%, with ICU-level utilization ranging from 0 to 91%. The five tertiary care hospitals accounted for 80% of the total ICU beds and 95% of all mechanically ventilated patients. Non-tertiary hospitals, which represent smaller urban, rural, and frontier coverage areas, had significantly higher rates on both adherence measures (*p* < .001) and represented 5% of patient encounters. Higher ICU-level use of the CDS tool in the provisional estimates was positively correlated with the percentage of patients receiving an initial low tidal volume strategy (*ρ* = 0.64, *p* = 0.024).

**Table 1:** Provisional estimates of low tidal volume and computerized protocol use by site

Sites	No. of ICUs	No. of ICU beds	No. of months CDS tool in place during the measurement period	Total no. of ventilated patient encounters during the measurement period	Estimated % encounters treated with initial set tidal volume ≤ 6.5 ml/kg PBW (%)	Estimated % encounters treated using at least one computerized protocol (%)
Site A*	5	84	3	316	80	59
Site B	1	13	11	40	68	83
Site C	1	4	7	24	63	79
Site D	1	4	7	18	61	50
Site E*	1	16	5	65	60	91
Site F	1	6	5	0	60	0
Site G*	1	16	13	753	52	50
Site H	1	6	8	25	52	64
Site I*	1	24	11	648	38	10
Site J	1	8	9	23	30	13
Site K	1	4	11	15	27	40
Site L*	2	38	17	984	21	6
Overall	17	223		2911	42	28
Tertiary hospitals (%)	59	80		95		
Tertiary hospitals only	10	178		2766	41	27
Non-tertiary hospitals	7	45		145	61	47
Absolute difference					20**	20**

*Tertiary site

**Significant at < .001

### Qualitative findings on ventilation and ventilator management practices

Forty-seven key informant interviews were conducted and analyzed at three ICU sites with varying levels of adherence. Demographic characteristics by role and by site are shown in Table 2. Saturation was reached with each role at each site. Initial adherence estimates were inconsistent with comments from key informant interviews that an initial set tidal volume of ≤ 6.5 ml/kg PBW was the standard practice for patient care at all three sites and that physicians consistently ordered the LPV CDS protocols for all mechanically ventilated patients. RTs reported that of the four protocols, the oxygenation and ventilation protocols were the most commonly used in actual practice. Based upon qualitative analysis of the 12 nurse interviews, the principal finding was that nurse exposure to LPV management and use of the LPV CDS tool was limited to coordinating with the RT on nurse-related aspects of care, such as sedation management, at all three sites. Nurses consistently stated they had no visibility to barriers and facilitators to LPV and LPV CDS use with limited understanding or influence regarding ventilation management activities. Nurses consistently reported that ventilation management was the purview of the physician and RT. Given this, the results below focus on physician and RT implementation barriers only.

**Table 2:** Key informant interview participant demographics

Participant characteristics	High-adhering site	Percent	Moderate-adhering site	Percent	Low-adhering site	Percent	Total	Percent
Sex								
Male	5	29	11	61	8	67	24	51
Female	12	71	7	39	4	33	23	49
Role								
Intensivist	3	18	7	39	4	33	14	30
Respiratory therapist	9	53	6	33	6	50	21	45
Nurse	5	29	5	28	2	17	12	25

### Implementation barriers and facilitators—individual/clinician (Table 3)

Physicians at all three sites were able to recall the key criteria for the diagnosis of ARDS. Identification of low-to-moderate severity ARDS cases, shown to be a challenge in prior studies, remained so for physicians and RTs in this study. Since non-ARDS mechanically ventilated patients were similarly started with an initial tidal volume setting of ≤ 6.5 ml/kg PBW, physicians felt that the detection of ARDS was less important to initiate a low tidal volume strategy. Once mechanical ventilation was initiated, clinicians at the low adherence site more frequently described returning to alternative ventilation and oxygenation strategies that they have used in the past versus applying LPV and LPV CDS tool instructions to non-responsive patients.

We’re happy to try it. But if it doesn’t work, we’re not going sit and watch our patient languish when we have something in our back pocket that in our experience has worked fine. Physician, low-adhering site

What we’ve found is… sometimes we’re playing catch-up…For us it’s hard to keep at a high FIO_2_, at a high PEEP, for X number of days, when in the past we’ve had huge changes and differences as soon as we’ve swapped to an open lung strategy. RT, low-adhering site

**Table 3:** Qualitative content analysis of key informant interviews—individual Consolidated Framework for Implementation Research domain

Qualitative content analysis themes	Clinician type	Relevant topic	Site adherence
Facilitator			
Ability to describe factors associated with the diagnosis of ARDS.	P	LPV	M, H
Detection of ARDS is not required to initiate low tidal volume or the protocols.	P	LPV, CDS	L, M, H
High confidence in the ability to use the underlying LPV CDS tool technology for patient care.	RT, P	CDS	L, M, H
Intent to use LPV and the LPV CDS tools	RT, P	LPV, CDS	L, M, H
Barrier			
Difficulty detecting low-to-moderate or developing ARDS in patients.	P	LPV	L, M, H
Return to alternative ventilation and oxygenation strategies that they have been successfully using in the past.	RT, P	LPV	L, M
Uncertain regarding the purpose and use of each of the four protocols in clinical care.	RT	CDS	L, M
Uncertain when its acceptable to depart from protocol recommendations.	RT	CDS	L, M

Clinician type—respiratory therapist (RT) and physician (P); relevant topic—lung-protective ventilation (LPV) and clinical decision support (CDS) tool; site adherence—low (L), medium (M), and high (H)

At the low and moderate adherence sites, uncertainty regarding the purpose and use of each of the four LPV CDS tool protocols, including when it was acceptable to depart from the protocol recommendations, was experienced as impinging on clinician self-efficacy. Despite the underlying variation in adherence, clinicians at all sites expressed confidence in their ability to use LPV and LPV CDS tool technology and expressed the intention to use them to deliver patient care.

### Implementation barriers and facilitators—intervention (Table 4)

While clinicians at the low- and moderate-adhering sites resented being told to adopt an intervention that they did not assist in selecting and developing, physicians at all sites felt that the use of the LPV CDS tool provided certain advantages relative to no computerized protocol. RTs at the high-adhering site felt that the use of the tool actually increased their self-efficacy when implementing an LPV strategy for ARDS patients. Critical care physicians and RTs agreed that an LTV setting was beneficial for patients with ARDS, but there was some disagreement regarding the tidal volume threshold for LTVV. Clinicians at the low-adhering site frequently identified patient-ventilator dis-synchrony or inadequate oxygenation as a reason to depart from LPV.

I’m always trying to adapt the ventilator as much as possible to the work and breathing of the patient…and trying to stay within their protocols of six mils per kilo. But sometimes it’s extremely difficult and near impossible. I worry because I see that patient retracting and fighting against the ventilator and I see more lung injury occurring. RT, low-adhering site

**Table 4:** Qualitative content analysis of key informant interviews—intervention Consolidated Framework for Implementation Research domain

Qualitative content analysis themes	Clinician type	Relevant topic	Site adherence
Facilitator			
Agreement that low tidal volume strategies are most appropriate for ARDS patient care	RT, P	LPV, CDS	L, M, H
Perception that use of a protocol provides certain advantages when treating patients with ARDS	P	CDS	L, M, H
Agreement that LPV CDS tool is easy to use once trained	RT, P	CDS	L, M, H
Belief that use of the protocols reduces physician time on ventilation management activities	P	CDS	L, M, H
Belief that LPV CDS tool use increases self-efficacy and confidence implementing an LPV strategy treating ARDS patients	RT	CDS	H
No need for significant changes in the technology design of the LPV CDS tool	RT, P	CDS	L, M, H
Barrier			
Resentment to adopting a care process they did not assist locally in selecting and developing	RT, P	LPV, CDS	L, M
Belief that LPV strategies can include initial tidal volume settings > 6.5 ml/kg PBW	RT, P	LPV	L
Perception that patients sometimes cannot tolerate low tidal volumes (patient-ventilator dis-synchrony)	RT, P	LPV	L, M
Belief that use of the tool increases time spent on documentation activities for each patient	RT	CDS	L, M
Discomfort with specific instructions from the LPV CDS tool given patient’s circumstances	RT, P	CDS	L, M
Perception that LPV CDS tool use does not facilitate a quick and efficient response to patient needs	RT	CDS	L, M
Perception that it is not easier to initiate a low tidal volume setting using the LPV CDS tool versus no tool	RT	CDS	L, M, H

Clinician type—respiratory therapist (RT) and physician (P); relevant topic—lung-protective ventilation (LPV) and clinical decision support (CDS) tool; site adherence—low (L), medium (M), and high (H)

This misperception regarding LPV appears associated at least in part with a misunderstanding regarding the role and use of PEEP strategies in LPV.

As intervention facilitators, RTs, and critical care physicians felt that the LPV CDS tool was easy to use after training and that major changes in the technology were unnecessary. Critical care physicians noted the benefit of being able to place a single order to facilitate patient ventilation management with change orders only required on an exception basis for significant departures from the LPV CDS protocols.

I really love the protocols. I worked at the University…and I can see the difference between not having protocols and having protocols. [At the University], all the vent changes had to be actively managed by the physicians…and it takes up a huge amount of time…it allows the RTs to really just run the protocol. Physician, high-adhering site

RTs at the high-adhering site felt that the LPV CDS tool actually made them more responsive to their patient’s needs when considering long-term patient health.

The way that we do vent checks every two hours…makes a big difference. We’re in the patients room more often, having eyes on the patient and seeing what’s going on…and then with our experience in the protocols, I think it does help us make those decisions in a faster way to wean patients and get them off the vent. RT, high-adhering site

In contrast, RTs at the low and moderate adherence sites felt that the use of the LPV CDS protocols slowed their response to patient needs and increased their workload. More experienced RTs noted that the use of the LPV CDS tool was not necessary to adhere to an initial tidal volume setting ≤ 6.5 ml/kg, which was inconsistent with the correlation observed in the quantitative data between LPV CDS tool use and initial tidal volume setting. More experienced clinicians felt that following the LPV CDS tool instructions was not always necessarily useful in making optimal care decisions for patients.

Most of the time…it is a high PEEP protocol. And so let’s say they’re on a PEEP of 20. It might prompt me to go down to 18. But I know that in two hours, I’m going to run this again, and it’s going to tell me again to wean the PEEP. But with my experience, I don’t like to wean the PEEP that fast… And when I have done that, I’ve seen them go backwards, or I’ve seen bad things happen and so I’m a little nervous too, I guess. RT, moderate-adhering site

Just the timeframe. If you run it every two hours and you’re seeing changes that need to be made…In most situations, it’s fine. But if you actually have a patient who is really sick, ARDS can progress really, really quickly…It’s frustrating to be in a situation where you know if you follow [the LPV CDS tool], you’re going to be four hours behind the eight-ball. RT, low-adhering site

### Implementation barriers and facilitators—organization/inner setting (Table 5)

Clinicians at the low-adhering site resented the perception that the use of LPV and the LPV CDS tool was a system-level mandate that limited their autonomy to select ventilation and oxygenation strategies.

We’ve never been asked. We’ve never been surveyed. We’ve never been talked to. We’ve been told. Physician, low-adhering site

**Table 5:** Qualitative content analysis of key informant interviews—inner setting Consolidated Framework for Implementation Research domain

Qualitative content analysis themes	Clinician type	Relevant topic	Site adherence
Facilitator or barrier			
Perception that use of LPV and the LPV CDS tool is a system-level, top-down mandate	RT, P	LPV, CDS	L, M, H
Frequent discussions between team members regarding the use of the LPV CDS protocols	RT	CDS	L, M, H
Barrier			
Resentment that clinicians have limited autonomy regarding selection strategies for specific patients	RT, P	LPV	L, M
Lack of clarity at all organization levels about the definition of success using LPV and LPV CDS tool	RT, P	LPV, CDS	L, M
Local site clinical leaders take limited accountability for improvement	RT, P	LPV	L
Local opponents had a meaningful impact on general attitudes regarding the system approach to LPV and the LPV CDS tool	RT, P	LPV, CDS	L
Limited formal training was provided to assist users in using the protocols correctly	RT, P	CDS	L, M, H
Perception that there was no conveniently accessible place to obtain consistent answers to questions on LPV CDS tool	RT	CDS	L, M
Perception that additional lab tests are required to run the LPV CDS tool	RT	CDS	L

Type—respiratory therapist (RT) and physician (P); relevant topic—lung-protective ventilation (LPV) and clinical decision support (CDS) tool; site adherence—low (L), medium (M), and high (H)

The lack of agreement both within and across sites regarding LPV adherence standards was itself a barrier to adherence. Clinicians felt a limited degree of accountability to achieve high adherence without a clear understanding of what constitutes high adherence. The lack of a clear definition of success impacted perceptions of the LPV CDS tool, particularly for more influential, experienced RTs who were familiar with earlier iterations of the tool or with the use of paper-based protocols.

At low-adhering sites, some more experienced RTs who had this view actively opposed both routine and consistent applications of LPV as well as utilization of the LPV CDS tool. These “anti-champions” often described being historically given substantial autonomy for ventilator management and tended to feel the LPV CDS tool reduced their autonomy. Informant interviews suggested these clinicians’ views were influential, impacting general attitudes among both RTs and physicians and low and moderate adhering regarding the benefits of LPV and the LPV CDS tool. Limited formal training to assist protocol users, including having convenient, accessible resources to obtain answers to questions, exacerbated frustrations.

While physicians did not indicate discussing the LPV CDS tool frequently with their peers, physician discussions with RTs and between RTs were commonly reported. Primary reasons for discussions centered on understanding and responding to instructions generated by the tool. Under the process, the RT needs to log into the EMR to conduct the ventilation assessment. Once the required ventilation assessment is conducted, the data is automatically loaded into the ventilator protocol. The RT is then required to open another page and click for instructions and then read and accept or reject generated instructions. Reasons for declining instructions are documented by the RT using a drop-down list. Perceptions of RTs from low-performing sites were that the additional steps and documentation in the workflow took too much time. This was particularly true when they had to repeatedly reject an instruction with each ventilation assessment.

### Impact of the initial approach to LPV CDS tool implementation (Table 6)

Strategies implemented during the initial rollout led to a meaningful increase in adherence rates for both LPV and the LPV CDS tool but were not sufficient alone to achieve high adherence. Sustainment efforts following the initial rollout at each site included ongoing executive leadership emphasis in team and organization meetings and site-level audit and feedback reporting. The initial LPV CDS tool implementation involved the rollout of all four components in a phased manner by individual site following the implementation of the new EMR. At the point of this study initiation, the phased rollout was complete and all 4 CDS tool protocols had been in place from 3 to 17 months, depending on the site. Users struggled to disentangle their sentiments about the LPV CDS tool itself from the impact on LPV CDS tool users’ experience of system downtime and network delays during the broader EMR rollout. There was a system RT champion available on site during the LPV CDS tool implementation and a system physician champion available by phone. The lack of an organized effort to discern from front-line clinicians what the barriers were, however, impaired the implementation team’s ability to understand and address concerns. Adherence data was available by site but was only available with a 3–4-month lag, limiting transparency to site and physician-level performance.

**Table 6:** Qualitative content analysis of key informant interviews—implementation Consolidated Framework for Implementation Research domain

Qualitative content analysis themes	Relevant topic
Facilitator or barrier	
Initial implementation strategies led to meaningful increases in adherence rates for both LPV and the LPV CDS tool. However, the strategies were not sufficient alone to achieve high adherence.	LPV, CDS
Barrier	
Initial implementation of the LPV CDS tool occurred as a small part of a large electronic medical record rollout.	CDS
No organized effort to discern from front-line employees what the barriers were to use.	LPV, CDS
No formally appointed implementation leaders were trained at each site.	LPV, CDS
Adherence data only available with a 3–4-month lag.	LPV, CDS
Deviations from the standards were not transparent to clinicians.	LPV, CDS

Relevant topic—lung-protective ventilation (LPV) and clinical decision support (CDS) tool

## Discussion

This study provides a rich understanding of implementation barriers and facilitators to the use of LPV and an LPV CDS tool in ICUs in an integrated system that was an early innovator in the use of LPV strategies, but has yet to achieve consistent high system adherence. This study’s main findings revealed multi-factorial facilitators and barriers to use that varied by ICU site adherence level. The primary facilitator was that LPV and the LPV CDS tool could be used on all mechanically ventilated patients. Barriers included a persistent gap between clinician attitudes regarding the use of LPV and actual use, the perceived loss of autonomy associated with using a computerized protocol, the nature of physician-RT interaction in ventilation management, and the lack of clear organization measures of success.

While clinicians consistently agreed that a set tidal volume ≤ 6.5 ml/kg PBW was optimal for patient care, we found more variation than expected when clinicians were asked to define LTVV and the appropriateness of ventilator modes other than volume control—our system standard—for the delivery of low tidal volumes. Consistent with prior studies [12, 13], clinician’s failure to recognize less-severe ARDS cases likely impedes LPV implementation. However, clinicians did not differentiate ARDS and non-ARDS patients when ordering the protocols or initiating an initial set tidal volume ≤ 6.5 ml/kg PBW but applied the standard to all mechanically ventilated patients. The fact that clinical teams are not required to differentiate ARDS patients when making decisions to utilize the LPV CDS tool or to initiate an initial low tidal volume setting is an important finding that simplifies implementation. This becomes important in situations, such as the COVID-19 pandemic, where ICUs are experiencing high rates of ARDS and are likely to benefit from consistent use of LPV [36].

Consistent with earlier studies [11], physician and RT statements that LPV strategies were most appropriate for patients with ARDS and indicated that they were consistently ordered and that an initial LTVV was used were not consistent with the provisional adherence data. This attitude-behavior gap [37] between intent and actual practice is consistent with earlier studies demonstrating that clinician beliefs and perceived barriers to using LPV were not correlated with LPV initiation [11, 14]. Several factors may begin to explain this gap. Efforts to promote adherence continued between the time when we generated provisional measurement data and when field interviews were conducted. These findings also suggest that non-attitudinal barriers such as perceptions of control; structural elements, such as workflows; and normative influences, including perceptions by the care team regarding patient impact [12], may continue to limit the use of LPV. These findings should be addressed in the development of implementation strategies [38].

The perceived loss of autonomy associated with following the LPV CDS protocol tools was difficult for more experienced RTs at low-performing sites. Under earlier paper-based systems, more experienced RTs may have felt empowered to function without physician orders in certain circumstances. Under relationship models theory, the traditional interaction between the physician and RT is based upon an authority ranking dyad relationship, asymmetrically ranked in a linear hierarchy with the critical care physician as the authority [39, 40]. At the low-performing sites, the traditional interaction was supplanted over time by an informal, negotiated market pricing relationship between the physician and RT. The critical care physician sought to optimize time in exchange for empowering more seasoned RTs to act in the patient’s interest as the RT saw fit, without always obtaining a physician’s order. The development of this informal, mutually protective dyad made it difficult to isolate and address the actual root causes of low adherence. Given that positive ventilation management outcomes are associated with the successful interaction of the physician and the RT, implementation strategies that target joint determinants should be considered. For example, using encounter-level data to identify on an exception basis those dyads that have higher non-adherence rates and conducting simulation training with each dyad to ensure that the required interaction is reasonably scripted and understood.

The lack of a definition for successful adherence to LPV and use of the LPV CDS tool was perceived as a key barrier at low-adhering sites. Relevant and timely information about performance on intermediate outcomes provides transparency at both the team level and with accountable system leadership and improves performance [41]. Given the low-volume, high-complexity nature of patient care in the ICU [42], detailed encounter-level data enables local teams to review performance on individual cases and diagnose implementation barriers and facilitators in real-life settings [43]. The lack of a definition of success also impacted perceptions of the LPV CDS tool. Teams focused on achieving a high adherence to a low initial tidal volume did not see the LPV CDS tool as useful in achieving this goal. However, higher-adhering sites described a broader view of the LPV CDS tool use benefits that was embedded in the way they do things, introducing a shared language for treating ARDS and driving their teams toward standardization in practice.

This study had certain limitations by design. The study does not establish a clear causal relationship between specific implementation barriers and facilitators and adherence. Adherence data available to inform site selection was initially limited to two provisional measures of performance and included all mechanically ventilated patients undifferentiated on ARDS status. Inclusion of all mechanically ventilated patients was likely not a significant weakness, however, as clinicians at all three sites indicated that ordering the protocols and use of an initial low tidal volume setting was the routine goal for all mechanically ventilated patients. Given that LPV and the LPV CDS tool protocols are used on all mechanically ventilated patients, some comments regarding barriers and facilitators to use may have been referencing all mechanically ventilated patients and not ARDS patients alone.

We were not able to interview all individuals in each role at the three selected sites given practical considerations but relied upon local site leadership to identify people for the team to meet with on the day of the site visit. Efforts were made to identify individuals within each role that varied in terms of years of experience and attitudes and beliefs regarding LPV and the LPV CDS tool. Evidence of saturation at each site mitigated the risk that important perspectives were not captured. Further, the field interview team reviewed the site interview list at the beginning of each site visit and debriefed throughout the day to determine if additional individuals should be added.

The use of researchers employed by the delivery system may have impacted the results, highlighting potential strengths and challenges of conducting embedded research linked to healthcare improvement [44–46]. While clear communication regarding the purpose of the interviews was included in the consent process and clinicians appeared forthright, we cannot rule out the possibility that interview participants’ comments were influenced by the investigator’s institutional alignment. The investigators’ individual biases, including the employment relationship, may also impact results.

## Conclusions

Variation in adherence to LPV persists in ICUs that were early adopters of LPV. Multi-factorial strategies that address the distinctive implementation barriers and facilitators of the ICU environment at the individual, team, and unit level appear necessary to achieve high adherence. As part of an implementation plan, organizations should consider initiating low tidal volume ventilation on all mechanically ventilated patients, identifying and agreeing upon standard adherence measures and designing education strategies that address physician-RT interaction during the care process. These strategies represent a blueprint for a future hybrid implementation-effectiveness trial.

## Supplementary information

**Additional file 1.**

## Data Availability

All provisional quantitative data generated or analyzed during this study is included as aggregated in the published article in Table 1. The majority of qualitative data for this study is made available in the publication tables. However, the detailed interview dataset generated and analyzed during the current study is not publicly available due to individual privacy concerns.

## Abbreviations

ABG: Arterial blood gas
ARDS: Acute respiratory distress syndrome
CDS: Clinical decision support
CFIR: Consolidated Framework for Implementation Research
ED: Emergency department
EMR: Electronic medical record
FiO_2_: Fraction of inspired oxygen
ICU: Intensive care unit
Intermountain: Intermountain Healthcare
LPV: Lung-protective ventilation
LPV CDS tool: Lung-protective ventilation clinical decision support tool
LTVV: Low tidal volume ventilation
PBW: Predicted body weight
PEEP: Positive end-expiratory pressure
RT: Respiratory therapist
UTAUT: Unified Theory of Acceptance and Use of Technology
