# Supplementary material for: Multi-factorial barriers and facilitators to high adherence to lung-protective ventilation using a computerized protocol: a mixed methods study
Source: Implement Sci Commun. 2020 Jul 28;1:67. doi: 10.1186/s43058-020-00057-x (PMC7385713; doi:10.1186/s43058-020-00057-x)
Supplement: Supplementary file 1 — Additional file 1. [file 43058_2020_57_MOESM1_ESM.docx]

**USE OF LUNG-PROTECTIVE VENTILATION FOR MECHNICALLY VENTILATED PATIENTS WITH ACUTE RESPIRATORY DISTRESS SYNDROME**

To begin, please share with us your organization role and brief clinical background as it relates to the treatment of mechanically ventilated patients with acute respiratory distress syndrome (ARDS).

**ARDS Detection and General Ventilation/Oxygenation Preferences**

1. Describe the criteria that you or your care team currently uses to identify a patient with acute respiratory distress syndrome (ARDS). What challenges exist for you or your care team in the correct identification of patients with ARDS?
2. When addressing the needs of a patient with ARDS requiring mechanical ventilation, what alternatives are available for the care team for ventilator management and oxygenation?
   1. What factors are important considerations for the care team when deciding on the ventilation and oxygenation approach and settings for each given patient?
   2. If the individual does not mention the use of lung-protective ventilation (LPV) or low tidal volume ventilation (LTVV) or high peak end expiratory pressure (PEEP)/fraction of inspired oxygen (FiO2) paired titration or ladder, ask them if they are familiar with or have used any of these approaches and how.

**Use of the LPV Computerized Protocols**

1. Describe your interaction to date with the LPV computerized protocols.
2. Which of the four LPV computerized protocols (ventilation, oxygenation, weaning assessment and pressure support (PS)/continuous positive airway pressure (CPAP)) do you currently use in patient care?

**Use of the LPV Computerized Protocols**

1. Do you find the LPV computerized protocols useful for you or for the care team in making optimal care decisions regarding the treatment and management of ventilated patients with ARDS?
   1. What specific protocols and features increase usefulness? What specific protocols features limit usefulness?
2. Do the LPV computerized protocols enable you or the care team to respond to your patient needs more quickly and more efficiently? Why or why not?
3. Does the LPV computerized protocols make it easier for you or for the care team to initiate LPV strategies for ARDS patients over the use of more traditional tidal volume settings? Why or why not?
4. How easy is it to use the LPV computerized protocols to manage LPV strategies in ARDS patients?
5. What changes would you make to the ventilation, oxygenation, weaning or spontaneous breathing components of the computerized protocol itself to improve usability?
6. How did you initially learn about each of the LPV computerized protocols?
7. Have you discussed the LPV computerized protocols with your peers or with anyone else, including your care team? If yes, please describe the circumstances and which protocol was discussed.
8. To what extent have you encountered disagreement within the care team on the use of LPV or the LPV computerized protocols? If meaningful, what was the source and primary reasons for disagreement?
9. How confident are you in your ability to use the LPV computerized protocols to effect change in treatment of mechanically ventilated patients with ARDS, if appropriate?
   1. *[If low confidence]* Do you have resources that could help you use the LPV computerized protocol to determine what, if any, appropriate actions might be taken?
   2. *[All]* What other information or tools do you need that you don’t have today that would help you effect change in your use of LPV for mechanically ventilated patients with ARDS?
10. Do you intend or plan to use the LPV computerized protocols for patients with ARDS in the near future? Why/why not? If you plan to use, how do you plan to use them?

**Conclusion**

1. Are there any questions we should have asked regarding LPV or the LPV computerized protocols that we didn’t? If so, what are they?
2. Do you have any other comments, feedback, or concerns about LPV or the LPV computerized protocols that you would like to share with us?
